# Herbal-Based Formulation Containing *Eurycoma longifolia* and *Labisia pumila* Aqueous Extracts: Safe for Consumption?

**DOI:** 10.3390/ph14020142

**Published:** 2021-02-10

**Authors:** Bee Ping Teh, Norzahirah Ahmad, Elda Nurafnie Ibnu Rasid, Nor Azlina Zolkifli, Umi Rubiah Sastu@Zakaria, Norliyana Mohamed Yusoff, Azlina Zulkapli, Norfarahana Japri, June Chelyn Lee, Hussin Muhammad

**Affiliations:** 1 Herbal Medicine Research Centre, Institute for Medical Research, National Institutes of Health, Ministry of Health Malaysia, Shah Alam 40170, Selangor Darul Ehsan, Malaysia; norzahirah.a@moh.gov.my (N.A.); elda@moh.gov.my (E.N.I.R.); azlina.zolkifli@moh.gov.my (N.A.Z.); umi.rubiah@moh.gov.my (U.R.S.); liyanaysf@ymail.com (N.M.Y.); farahjapri15@gmail.com (N.J.); june_lee@moh.gov.my (J.C.L.); hussin.m@moh.gov.my (H.M.); 2 Medical Resource Research Centre, Institute for Medical Research, Jalan Pahang, Kuala Lumpur 50588, Wilayah Persekutuan Kuala Lumpur, Malaysia; azlina.zulkapli@moh.gov.my

**Keywords:** *Eurycoma longifolia*, *Labisia pumila*, tongkat ali, kacip fatimah, toxicity

## Abstract

A combined polyherbal formulation containing tongkat ali (*Eurycoma longifolia*) and kacip fatimah (*Labisia pumila*) aqueous extracts was evaluated for its safety aspect. A repeated dose 28-day toxicity study using Wistar rats was conducted where the polyherbal formulation was administered at doses 125, 500 and 2000 mg/kg body weight to male and female treatment groups daily via oral gavage, with rats receiving only water as the control group. In-life parameters measured include monitoring of food and water consumption and clinical and functional observations. On day 29, blood was collected for haematological and biochemical analysis. The rats were necropsied and the organs were collected for histopathological examination. This study showed that the combined formulation did not induce any significant toxicity effect at any dose level in terms of morbidity, mortality, behaviour, functional observation, body weight, food and water consumption, whole blood haematology and serum biochemistry. However, there were some microscopic changes in the histopathological examinations of some organs given 2000 mg/kg body weight, which may suggest an early response to the polyherbal formulation. From this study, the no observed adverse effect level is estimated to be more than 500 mg/kg body weight but not exceeding 2000 mg/kg body weight. The observed effects at the highest dose indicate the need for further study of longer dosing duration.

## 1. Introduction

Traditionally, medicinal plants have been used in disease prevention and treatment for many generations and their potential pharmaceutical values have easily drawn worldwide attention. At the global stage, the World Health Organization (WHO) has launched the WHO Traditional Medicine Strategy 2014–2023 to reinforce the use of medicinal plants in healthcare services and management [1]. Consumers at present are conscious about growing price [2] and frequent adverse events often associated with conventional drugs [3]. Consequently, the trend towards maintaining health and wellbeing using medicinal plants as an alternative treatment is expanding. However, some medicinal plants have been reported to trigger allergic reactions [4,5], modify bioavailability of conventional drugs [6,7,8] and cause organ toxicity such as hepatotoxicity, nephrotoxicity and cardiotoxicity [9,10,11]. Therefore, the consumption of medicinal plants merely based on traditional practices that lack valid scientific evidence may overrule their benefits due to these possible undesirable health effects. Evidence-based safety studies investigating medicinal plants and the products thereof are beneficial to both the scientific community for pharmaceutical development, as well as to the consumers in seeking alternative plant-based therapies.

Root aqueous extracts of *Eurycoma longifolia* Jack (family Simaroubaceae, commonly known as tongkat ali) and roots or whole plant aqueous extracts of *Labisia pumila* (Blume) Fern-Vill (family Primulaceae, kacip fatimah), which are native plants of Southeast Asia, are two popular choices of herbs consumed in this region [12]. Tongkat ali roots have been traditionally used to improve various health conditions such as fever, boils, wounds, ulcer, infertility, bleeding gums, aches, dysentery, glandular swelling, edema and as an afterbirth medication and tonic [13,14]. The whole plant of kacip fatimah is used but mainly the roots are used traditionally to aid in the labour process of contraction and induction, help regain body strength after giving birth and relieve other health problems such as flatulence, dysentery, dysmenorrhoea and gonorrhoea [15,16]. The scientific investigations into these claims looked into different extraction processes of these main plant parts and largely explored tongkat ali’s effect on male fertility [17,18,19,20,21,22,23,24] and its antimalarial action [20,25,26,27,28,29], while kacip fatimah has been tested for its potential in women’s reproductive health [30,31,32,33,34,35] and, to some extent, their cytotoxic [35,36], antimicrobial [37,38], anti-inflammatory [30,39], antioxidant [39,40] and anti-osteoporosis [41,42,43,44] capacities. Improvements in male sexual health is evidenced by improvements in seminal and fertility parameters, as well as increased testosterone levels in in vivo models and clinical trials, largely attributed to its quassinoid content [45,46,47]. The phytoestrogenic potency of kacip fatimah shown by the suppression of proinflammatory cytokines (responsible for osteoporosis) combined with its high scavenging properties (for reducing oxidative stress), regulating hormonal changes and providing relief of post-menopausal symptoms [30,48]. Although there is no knowledge of both herbs used in combination traditionally, the use of these two herbs in combination is of scientific interest as they show potential health benefits in different organ systems.

The idea of using combinatorial herbal formulations is an age-old method in traditional medicines with the aim to boost the effects of the combined herbs in a synergistic manner [49,50]. However, along with the benefits of these polyherbal formulations, some harmful effects may also be exacerbated through a combined formulation [51]. Both the aqueous extracts of tongkat ali and kacip fatimah evaluated independently showed a high tolerance value of up to 5000 mg/kg body weight in rats [52,53]. However, Singh et al. (2009) reported the no observed adverse effect level (NOAEL) for their kacip fatimah extract to be 50 mg/kg [54].

The use of an individual NOAEL value as a step towards determining the safe starting dose in humans must be done with caution in a combination formulation [55,56]. The NOAEL value accounting for the combined formula and the effect of repeated exposure via safety testing using the specific formulation that accounts for all the compounds must be established. Some safety information of tongkat ali and kacip fatimah as individual herbal plants in their aqueous extracts for both plants, as well as methanolic extract and powdered form for tongkat ali, which were tested in the rat model and clinically (for tongkat ali), are available [57,58,59,60,61], however, the safety data for the combination of these two medicinal plants in any animal model have not been established. Previously, an acute oral toxicity study for the combined extracts was conducted in our facility, where the NOAEL value was determined to be more than 2000 mg/kg (unpublished work). Therefore, it is crucial to identify any health risks associated with the repeated use of this combined herbal formulation. In this study, the potential toxicity of a polyherbal formulation termed the P.SLP formulation, containing aqueous extracts from *E. longifolia* (roots) and *L. pumila* (whole plant), was evaluated in both genders of Wistar rats following daily oral administration for 28 days.

## 2. Results

### 2.1. Chemical Identification of Test Item (P.SLP Formulation)

**Figure 1:**
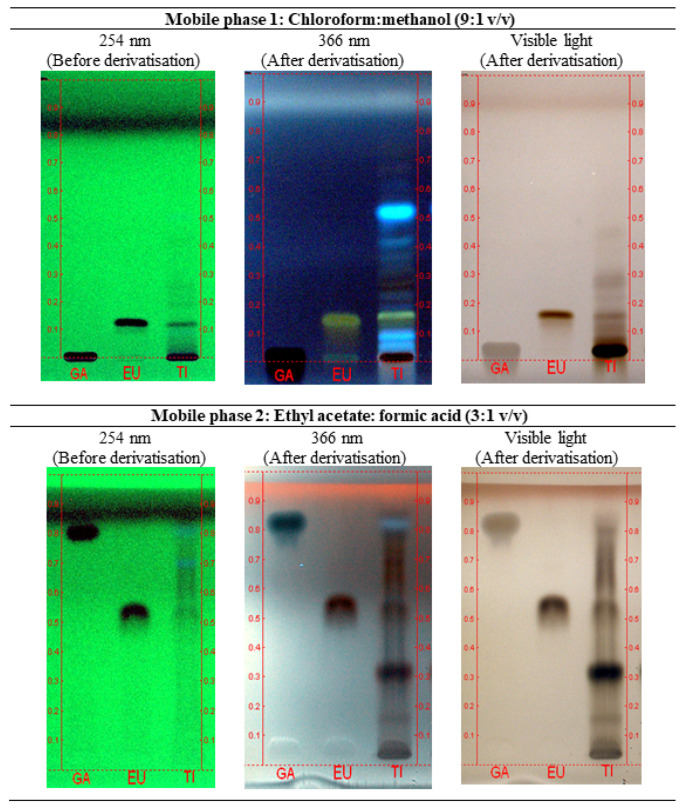
High-performance thin layer chromatography (HPTLC) profiles of gallic acid (GA), eurycomanone (EU) and methanol solution of P.SLP formulation (TI) observed under 254 nm (before derivatisation), 366 nm and visible light after derivatisation with 10% sulphuric acid solution.

Chromatographic identification analysis is commonly used for the quality assessment and species authentication of medicinal plants, including herbal products such as health supplements. In this present study, high-performance thin layer chromatography (HPTLC) and liquid chromatography–mass spectrometry (LC-MS) methods were used to authenticate the P.SLP formulation containing aqueous extracts from *E. longifolia* and *L. pumila*. Eurycomanone and gallic acid were used as chemical markers to identify *E. longifolia* and *L. pumila*-based formulations. A mixture of chloroform:methanol (9:1 *v*/*v*) (as mobile phase 1 (MP1)) were used to detect eurycomanone, which is detected at a retention factor (R_f_) of 0.13 under ultraviolet (UV) 254 nm before derivatisation and under UV 366 nm (green zone) and under white light (violet zone) after derivatisation with 10% sulphuric acid solution. The MP 2, a mixture of ethyl acetate:formic acid (3:1 *v*/*v*) was used to detect both gallic acid at R_f_ of 0.81 and eurycomanone at R_f_ of 0.53 under UV 254 nm before derivatisation. The markers were also observed after derivatisation with 10% sulphuric acid under UV 366 nm (gallic acid at R_f_ of 0.81 (blue zone) and eurycomanone at R_f_ of 0.53 (violet zone)) and under white light (gallic acid at R_f_ of 0.81 (violet zone) and eurycomanone at R_f_ of 0.53 (violet zone)) (Figure 1).

**Figure 2:**
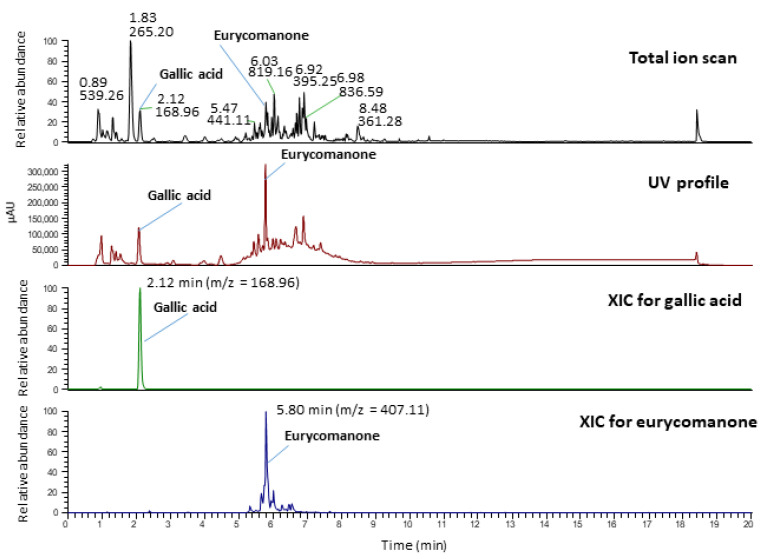
The LC-MS profile of the P.SLP formulation and the extracted ion mass of chemical markers gallic acid (retention time (t_R_) = 2.1 min, mass-to-charge ratio (*m*/*z*) = 169) and eurycomanone (t_R_ = 5.80 min, *m*/*z* = 407).

**Figure 3:**
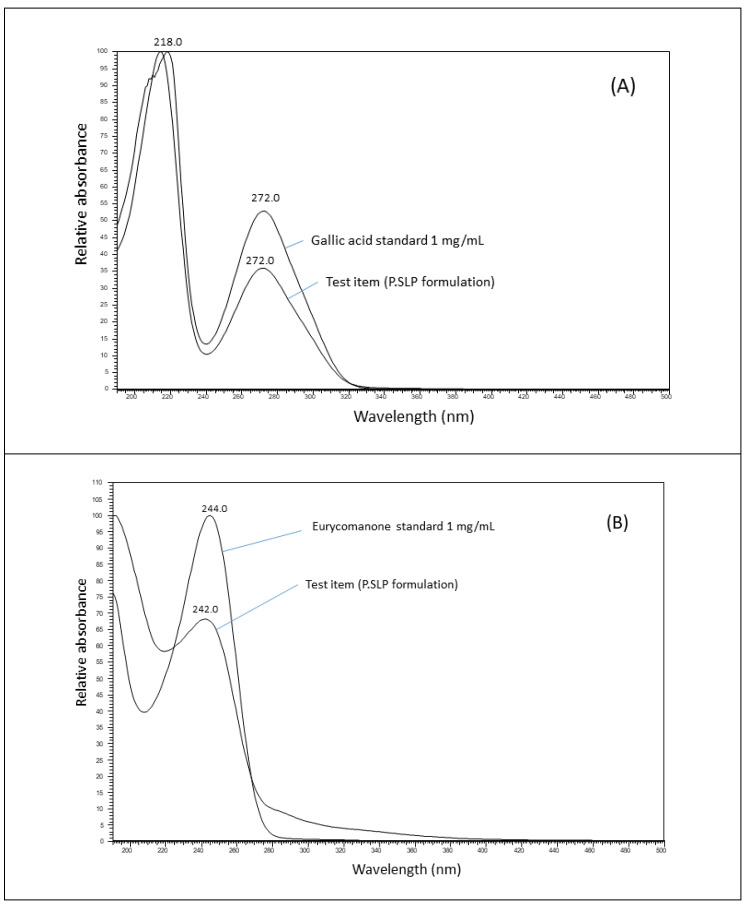
UV spectrum of (**A**) gallic acid standard (1 mg/mL) and (**B**) eurycomanone standard (1 mg/mL) overlaid with methanol solution of the P.SLP formulation.

Meanwhile, identification of the formulation was also evaluated on an LC-MS instrument coupled to a UV detector. The markers, gallic acid and eurycomanone were detected and identified based on mass-to-charge ratio (*m*/*z*), retention time (t_R_) and UV profile with reference to the chemical standards. The chromatogram of the formulation is shown in Figure 2 whilst its UV profile is shown in Figure 3. The LC-MS-UV results showed that the marker compounds gallic acid and eurycomanone were both detected in the sample: gallic acid [M-H]- of 169 (t_R_ = 2.12 min, UV_max_ = 272 nm) and eurycomanone [M-H]- of 407 (t_R_ = 5.80 min, UV_max_ = 244 nm).

### 2.2. Mortality, Clinical and Functional Observations

No morbidity or mortality were observed in any rats, except for a female rat in the high-dose satellite group that was found dead on day 28 of dosing. The post-mortem findings (data not shown) indicated the cause of death was due to technical error and not formulation related. No clinical sign of toxicity was observed in the any P.SLP formulation-treated or control groups (Table 1).

No significant difference was found in the general activity, locomotor and stereotyped movements for all groups. However, the forelimb grip strength of female rats in the medium- and high-dose groups was shown to be significantly stronger compared to the vehicle control group (Table 2).

### 2.3. Body Weight

The body weights gradually increased each week for all groups except for a slight decrease (0.59%) in the sixth week in the male vehicle control satellite group. However, there was no significant difference in the percentage weekly body weight change between P.SLP formulation-treated groups and their respective vehicle control groups (Table 3).

### 2.4. Food and Water Consumption

All rats had normal food and water consumption (94.45–187.36 g/week; 197.2–694.8 mL/week) throughout the study. No significant difference in the percentage of weekly food and water consumption between any P.SLP formulation-treated groups and their respective control groups was found in either gender (Table 4 and Table 5).

### 2.5. Haematology and Clinical Biochemistry

Administration of the P.SLP formulation showed no significant effect on haematocrit (HCT), haemoglobin concentration (HGB), erythrocyte count (RBC), total leukocyte count (WBC) and platelet count (PLT) when compared to the respective control groups (Table 6). No significant difference was found in potassium (K), glucose (GLUC), total cholesterol (CHOL), urea (BUN), creatinine (CREA), total protein (TP), albumin (ALB), triglyceride (TGL), uric acid (URCA), alanine aminotransferase (ALT), alkaline phosphatase (ALP) and aspartate aminotransferase (AST) levels when compared to the respective control groups (Table 7 and Table 8).

### 2.6. Gross Pathology Examination

Gross lesions were found in the lung, intestinal tract, liver, kidneys, ovaries and uterine horn across all dose groups (Table 9). Other macroscopically examined organs such as the heart, spleen, stomach, adrenals and testes did not show any abnormalities.

### 2.7. Relative Organ Weight

There was no significant difference in the organ weight relative to 100 g body weight in either gender for all organs other than the left ovary. A significantly lower relative weight was observed for the left ovary in the low-dose (0.0188), medium-dose (0.0166) and high-dose satellite (0.0183) groups compared to their respective control groups (vehicle control: 0.0256, vehicle control satellite: 0.0205) (Table 10 and Table 11).

### 2.8. Histopathology

There were some microscopic findings on the histological appearance of the organ tissues (liver, kidneys, adrenals, testes, ovaries, uterine horn, spleen, lungs, heart, stomach and intestinal tract) for low and medium groups, but it was not a P.SLP formulation-related toxicity effect. However, the heart, lung, liver, spleen, kidneys, stomach, intestinal tract, ovaries and adrenal from the high-dose groups (five females, four males) and the high-dose satellite groups (four females, five males) were found to have some histological observations indicating an early response to exogenous toxicity (Table 12).

**Figure 4:**
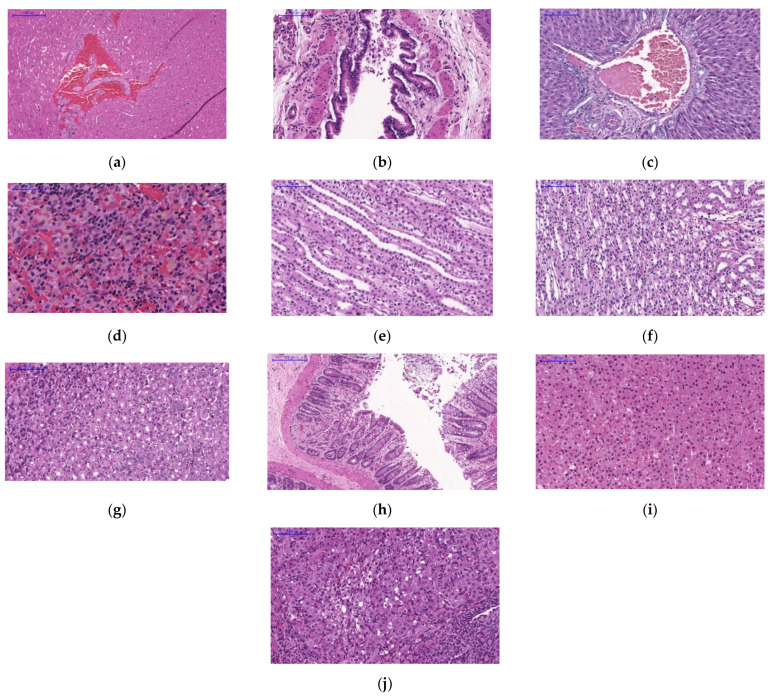
Histological specimens of rats’ tissues were collected after 28 days of treatment. Tissue samples were stained with haematoxylin and eosin. Representative histological pictures from the 2000 mg/kg dose groups were taken at the following magnifications: (**a**) heart (10×), (**b**) lung (20×), (**c**) liver (20×), (**d**) spleen (40×), (**e**) left side of kidney (10×), (**f**) right side of kidney (20×), (**g**) stomach (20×), (**h**) intestinal tract (10×), (**i**) left side of adrenal (20×) and (**j**) right side of ovary (40×).

In the 2000 mg/kg dose-treated groups, some sections of the heart showed cell degeneration, congestion and hyaline tissue degeneration in the blood vessels and the muscle fibres were slightly pale and less dense. The lung tissues showed bronchial infiltration with epithelial and red blood cells. Hepatocytes were found swollen and the central veins were congested with blood. Mild depletion of lymphoid cells was observed in sections of spleen. Sections of kidneys showed tubular hyalinisation and degeneration of tubule epithelial cells within the renal medulla region. Sections of stomach showed cell degeneration and depletion of cytoplasmic granules and there was also vacuolated villous epithelium found in the sections of intestinal tract. Swelling and degeneration of cytoplasmic cells were found in the adrenal sections. Cytoplasmic vacuolation was observed in the sections of the ovaries (Figure 4).

## 3. Discussion

Prediction of the adverse effects of the polyherbal formulation in humans over a range of doses, dosage regimens and exposure durations by means of the rat model is the main goal in this current study. Repeated daily oral administration of the P.SLP polyherbal formulation for 28 days in rats is commensurate with its repeated exposure for 2.7 years in humans [62]. Other than being inexpensive and easy to maintain, the rat model was chosen as it is one of the recommended and preferred rodent species by regulatory authorities, as it is a sufficiently characterised species, with high sensitivity in expressing any toxic responses [63]. The Wistar rats (*Rattus norvegicus*) were chosen in order to maintain a consistent animal model, and were similarly used in the previous 14-day single oral dose study (unpublished work). Clinical and functional observations, body weight changes, food and water consumption and blood parameters did not show any significant toxicity effects at any dose level. However, some findings, namely on one mortality (incidental death) and the relative ovary weight parameter in some dose groups, as well as findings in gross pathology examination and histology assessment, particularly in the high-dose group, are reported in this study.

There was one death in the female high-dose satellite group due to personnel technical error that may have led to gavage-facilitated reflux resulting in the spontaneous death [64]. The statistically significant low weights of the ovaries showed no clear dose relationship and there was a lack of correlation with histological findings, except in the high-dose satellite group. Hence, these two circumstances were not considered to be P.SLP formulation related.

Organs, including ovary in the high-dose and high-dose satellite groups, were found to show microscopic histology findings associated with exogenous toxicity. Exogenous toxicity is caused by a toxin that is externally presented to the body and may originate from the materials supplied to the rats, such as the food pellets, drinking water and bedding. Nevertheless, in this study, all materials supplied to the rats were certified to be safe for their respective use. Our previous 14-day single oral dose study showed no acute toxicity findings at any dose levels tested (5, 50, 300 and 2000 mg/kg body weight) (unpublished work), therefore, the repeated exposure of the P.SLP formulation at a high dose (2000 mg/kg) could account for the microscopic changes of organs in the high-dose groups.

Of the few publications on the repeated oral administration of tongkat ali extracts at 1000 mg/kg (aqueous extract), 2000 mg/kg (powdered root) and 2400 mg (aqueous extract) in rats, none reported any treatment-related mortality or clinical signs of toxicity [52,58,59]. A study on eurycomanone, which is a prominent chemical marker in *E. longifolia*, suggests that once it is absorbed in rodents, it is not easily metabolised and it can actively exert its pharmacological activities [65]. It is inferred that the low percentage of bioavailability, at approximately 11%, is unlikely to cause severe toxicity effects [65,66]. However, no steady state data are available to compare eurycomanone’s cumulative effect when administered in a repeated dose study of either the extract or the compound form.

As for the kacip fatimah aqueous extract, there is a 100-fold difference between the lowest reported NOAEL at 50 mg/kg and the highest reported tolerable dose at 5000 mg/kg [53,54]. This huge difference may indicate that these reported doses may not be the representative NOAEL doses for kacip fatimah aqueous extracts. A closer look at kacip fatimah’s active ingredient, gallic acid, showed that the repeated oral administration of gallic acid at 900 mg/kg in mice (calculated rat dose of approximately 450 mg/kg) did not elicit any toxicity [67]. Therefore, it is deduced that the presence of gallic acid in the P.SLP formulation at 0.07% is safe.

Ultimately, findings from this study are useful for determining the safe human dose for this combined formulation for its subsequent clinical use [68]. When extrapolating to the human equivalent dose (HED), the uncertainties associated with animal data are offset by the routine use of a 10-fold interspecies uncertainty factor [69,70]. The calculated HED for the high dose level (2000 mg/kg) by dividing by the safety factor of 10 is 32.25 mg/kg (equivalent to 1.94 g of the formulation taken by a 60 kg human). The recommended daily human dosage is two capsules (250 mg of P.SLP formulation per capsule) equivalent to 8.3 mg/kg body weight or 0.5 g P.SLP formulation intake by a 60 kg human [71]. Comparison between the calculated HED (1.94 g/60 kg human) and the recommended human dose (0.5 g/60 kg human) indicates that the highest tested concentration (2000 mg/kg) in this study is equivalent to 3.9 times more than the recommended intake in humans. In essence, the recommended dose level is four-fold lower than the highest tested dose level and corresponds to the medium dose level (500 mg/kg) tested in this current study. The medium dose level seems unlikely to reveal any P.SLP formulation-related toxicity response.

By contrast, some of the gross lesions detected in some organs during the gross examination had no P.SLP formulation-related toxicity signs when they were analysed microscopically. Mechanical injury, such as puncture holes due to the technical approach or physical pressure during organ harvesting or grossing, could have possibly caused these reported lesions. Consequently, pneumocytes within the interalveolar septa would compress one another and the extra pressure on the alveolar wall could have possibly caused rupture and losses of alveolar spaces [72]. The need for improved handling techniques and finer tools is critical to avoid any unwanted lesions on the organ tissues [73]. Another recorded observation includes the reddish colour of the uterine horn, which may be due to the respective female rats being “in heat” (oestrus cycle), resulting in the appearance of prominent blood vessels [74]. However, vaginal smear to confirm such an observation was not conducted in this study. The macroscopic lesions and microscopic findings on the lungs and heart were likely associated with the trauma from blood collection via cardiac puncture, which allowed maximum blood volume collection [75]. Lesions on the organ tissues were deemed accidental and unrelated to the formulation administration.

The use of isoflurane in this study is of minimal concern. Inhalation of isoflurane with oxygen as the carrier gas was used as the anaesthetic agent to produce the state of unconsciousness, thus preventing the animal from suffering. The blood solubility coefficient of the inhalation mode is lower than in the injection mode, which results in the animals taking a longer time to lose consciousness, compared to the injection mode [76]. However, the use of oxygen as the carrier gas in the inhalation mode improves tissue oxygenation during anaesthesia that maintains homeostasis and cellular metabolism in the tissues [77]. In addition, the metabolism of volatile-based anaesthetics including isoflurane is negligible and would interfere minimally with liver function and the metabolism of the test item in a study [78,79]. Hence, the effect of using isoflurane on the findings from this toxicology study is not significant.

The effects of the daily administration of doses at 125, 500 and 2000 mg/kg/day for 28 days consecutively, along with all the investigated experimental parameters in the rat model, were sufficiently assessed to obtain relevant toxicology data of the P.SLP formulation. The observed early exogenous toxicity effects at the highest dose indicate the need for further study of a longer dosing duration. Recently, extensive studies have been carried out on other combined herbs to further understand undesirable effects such as any potential interaction (synergistic or antagonistic) with other substances, such as food and conventional drugs [80,81]. Therefore, a similar approach for this polyherbal formulation should also be implemented where its efficacy or any tendency to cause specific organ toxicity is explored.

## 4. Materials and Methods

### 4.1. Test Item

The test item (P.SLP formulation) was provided by Biotropics Malaysia Berhad (762243-P) (Lot 21, Jalan U1/19, Section U1, Hicom Glenmarie Industrial Park, 40150 Shah Alam, Selangor Darul Ehsan, Malaysia). It contains, namely, Physta^®^ Tongkat Ali (trade name of *E. longifolia* plant aqueous extract) with a bioactive composition of 0.12% eurycomanone and SLP+^®^ Kacip Fatimah (trade name of *L. pumila* plant aqueous extract) with 0.07% gallic acid content (certificate of analysis number: BMB/P17/RE019).

### 4.2. Chemicals and Reagents

Chemical standard gallic acid (CAS no.: 84633-29-4) was purchased from Sigma-Aldrich (St. Louis, MO, USA) and eurycomanone (CAS no.: 149-91-7) from ChromaDex Corp. (Los Angeles, CA, USA). Solvents used such as methanol, chloroform, ethyl acetate, formic acid, sulphuric acid and acetonitrile were purchased from Merck & Co., Inc. (Darmstadt, Germany).

### 4.3. Chemical Identification of the Test Item

Chemical identification of the test item was obtained using the HPTLC and LC-MS techniques. The identity of *E. longifolia* and *L. pumila* in the formulation was verified using gallic acid and eurycomanone as the chemical markers [82,83].

The chemical standards gallic acid and eurycomanone were prepared at a concentration of 1 mg/mL in methanol. For the sample preparation, 1 g of the test item was dissolved in methanol and sonicated for 15 min. After sonication for 15 min, the test item solution was filtered through a 0.2 µm filter and used for the chemical identification analysis.

The HPTLC chromatography was performed on 10 × 10 cm HPTLC silica gel 60 F254 plates. Standard and test item solutions of 5 µL were separately applied to the plate as 8 mm wide bands with an automatic TLC applicator Linomat-V (CAMAG, Muttenz, Switzerland), 8 mm from the bottom. Two different mobile phases consisting of chloroform:methanol (9:1 *v*/*v*) (MP1) and ethyl acetate:formic acid (3:1 *v*/*v*) (MP2) were used per chromatograph. The plate was developed in a 10 × 10 twin glass chamber saturated with mobile phase at room temperature up to 7 cm. After development, the plates were air dried and then derivatisation of the chromatogram was performed by dipping the plate in 10% sulphuric acid in water, heated at 105 °C for 5 min or until the colour of the zones became visible. The plates were observed under the CAMAG UV cabinet at 254 nm (before derivatisation) and under UV 366 nm and white light (after derivatisation).

The LC-MS analysis was performed using a Dionex Ultimate 3000 UHPLC system (Thermo, Sunnyvale, CA, USA) coupled to a diode array detector and ion-trap mass spectrometer (Thermo, San Jose, CA, USA). Chromatography separation was carried out using an HSS T3 column (2.1 mm × 100 mm, 1.8 µm, Waters, Milford, MA, USA). Gradient elution was performed with 0.1% formic acid in water (A) and 0.1% formic acid in acetonitrile (B) at a flow rate of 0.3 mL/min. The initial condition was 5% B, which was held for 3 min and then increased to 100% B over 9 min, and kept constant at 100% B for 5 min. Finally, the initial condition was restored and held for 3 min to re-equilibrate the system. The total run time was 20 min. The column oven was maintained at 40 °C. The injection volume was 1 µL. The mass spectrometer was operated in electrospray ionisation (ESI) negative mode. Nitrogen was used as nebuliser gas at a pressure of 100 psi, as carrier gas at 15 L/min and 150 °C and as sheath gas at 35 L/min at 320 °C. Identification of chemical marker peaks was performed by comparison against the gallic acid and eurycomanone standard.

### 4.4. Preparation of Test Item for Animal Study

The P.SLP formulation was added to reverse osmosis water (vehicle) to achieve concentrations of 12.5, 50.0 and 200.0 mg/mL. The formulation was then administered orally once daily for a period of 28 days. The volume given was adjusted to the body weight of the rats and the pre-determined dosing volume of 10 mL/kg body weight. The oral route was used in this study as it is the recommended route for rats, as well as the intended route for human use.

### 4.5. Experimental Animals

Rat (*Rattus norvegicus*) model, i.e., Wistar rats was used in this study. Sixty Wistar rats (30 rats per gender, eight to nine weeks old upon exposure to the P.SLP formulation, weighing 210–220 g for females and 309–312 g for males) were obtained from Beijing Vital River Laboratory, Animal Technology Co., Ltd., No. 4 Yangshan Road, Chaoyang District, Beijing, 100107, P.R. China.

The rats were housed in individually ventilated cages with corn-cob bedding (Corn Cob Laboratory Bedding (Biocob corncob), Biosys Corporation Pte Ltd., 111 North Bridge Road, #27-01 Peninsula Plaza, Singapore 179098) for a 14-day quarantine period to monitor their health condition. Using the same housing system, the rats were then acclimatised to the laboratory condition and human handling for five days prior to the start of P.SLP formulation administration. The experimental room was maintained at a temperature of 19–26 °C and humidity at 35–65%, with a 12 h light–dark cycle. The rats were given standard rodent pellet diet (Specialty Feeds, 3150 Great Eastern Highway, Glen Forrest, Western Australia 6071) and an unlimited supply of reverse osmosis water. The handling of rats was performed in accordance with the animal handling guideline by the Ministry of Health Malaysia [84].

### 4.6. Animal Experimental Procedures

This study was conducted in accordance with test guideline 407 under the Organisation for Economic Cooperation and Development principles of good laboratory practice [68] at the Non-Clinical Research Facility, Good Laboratory Practice Section, Institute for Medical Research, Malaysia. Care and use of study animals were handled in compliance with the test facility’s standard operating procedures. From the findings of the single dose 14-day oral toxicity study (unpublished work), the NOAEL was more than 2000 mg/kg, therefore the dose 2000 mg/kg was selected as the highest dose in this study. The medium and low doses were calculated such that they were four-fold lower than the high and medium dose, respectively, as recommended by the test guideline. Therefore, the three dose levels used in this study were 125, 500 and 2000 mg/kg body weight.

**Figure 5:**
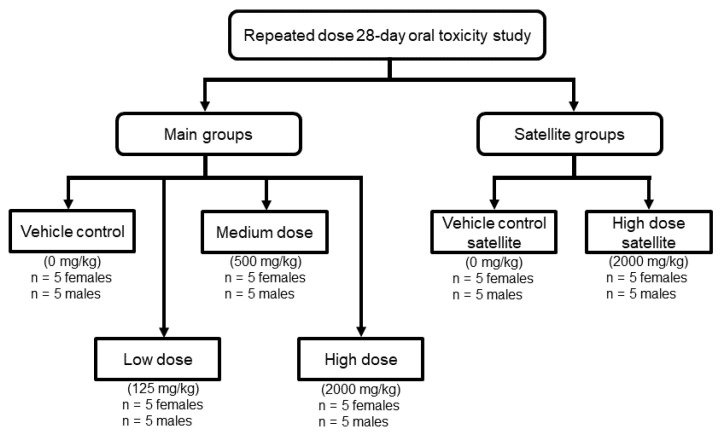
Study design for repeated dose 28-day oral toxicity study. Total number of rats involved was 30 females and 30 males.

For each gender, the rats were equally divided into six groups (five rats per group) randomly. The P.SLP formulation-treated groups were low-dose, medium-dose, high-dose and high-dose satellite groups, while the control groups were vehicle control and vehicle control satellite groups (Figure 5). Administration of the formulation was performed via oral gavage using a ball-tipped intubation needle fitted on a syringe. All the rats were administered with the formulation for 28 days, except for the satellite groups that were kept for an additional 14 days without dosing to detect delayed occurrence or persistence of, or recovery from, toxic effects.

On the final day of oral dosing, the feed pellets were removed to allow overnight fasting. The rats were necropsied on the next day. Each rat was anaesthetised using isoflurane (0.25–2.0% isoflurane with 0.5–2.0 L/min of oxygen, Veterinary Companies of Australia Pty Ltd., New South Wales, Australia) for blood sampling and exsanguination by abdominal aorta, as advised by the veterinarian. The euthanised rats were subjected to gross pathology examinations by the veterinarian. Any abnormality was recorded. The weighed organs were then subjected to gross and histopathological examination.

#### 4.6.1. Mortality, Clinical and Functional Observation

The rats were observed for morbidity and mortality twice daily, once in the morning and once in the afternoon, at approximately the same time. General clinical observations were conducted once daily two hours after dosing. Detailed clinical observations (changes in skin, fur, eyes and mucous membranes, occurrence of secretions and excretion, autonomic activity, changes in gait, posture and response to handling, clonic or tonic movements, stereotypies, bizarre behaviour and abnormalities of the oral cavity) were conducted once before the first exposure to the P.SLP formulation and once weekly thereafter. Functional observation was conducted during the fourth week of exposure in order to assess the rats’ grip strength using a grip strength meter (BIOSEB, Vitrolles, France) and motor activity (general activity, locomotor and stereotyped movements) using an actimeter (Actitrack, Panlab, S.L., Barcelona, Spain). The observation procedures were performed by the veterinarian and laboratory personnel.

#### 4.6.2. Body Weight

The body weight for each rat was measured prior to dosing, weekly thereafter and prior to necropsy. The weekly percentage body weight change was calculated.

#### 4.6.3. Food and Water Consumption

Each feed pellet packet was weighed and the amount of water in the drinking bottle was measured prior to placement in the cage. The leftover amounts of feed pellets and drinking water in the cage of each rat was recorded weekly. The consumptions for both were then calculated as a percentage.

#### 4.6.4. Haematology and Clinical Biochemistry

The collection of blood was carried out via cardiac puncture and abdominal aorta. Whole blood was transferred to ethylenediaminetetraacetic acid tubes (for the haematology analysis) as well as gel and clot activator tubes (for the clinical biochemistry analysis). The following parameters were analysed for each whole blood sample using a haematology analyser (CDS Medonic CA 620, Plantation, FL, USA): HCT, HGB, RBC, WBC and PLT. Serum samples were analysed using a clinical biochemistry analyser (Siemens Dimension^®^ Xpand Plus, Newark, DE, USA) for the selected parameters: K, GLUC, CHOL, BUN, CREA, TP, ALB, TGL, URCA, ALT, ALP and AST. The blood sampling procedures were conducted by the laboratory personnel and veterinarian.

#### 4.6.5. Gross Pathology Examination

All rats were subjected to a gross pathology examination of the body surface and subcutis on necropsy day. Initial inspection was also made on their organs (liver, kidneys, adrenals, testes, ovaries, uterine horn, spleen, lungs, heart, stomach and intestinal tract). All examination procedures were conducted by the veterinarian.

#### 4.6.6. Relative Organ Weight

The collected organs were cleaned of any adherent tissues or fats and weighed. All paired organs were weighed separately. The relative organ weight of the organ (organ weight relative to 100 g body weight of rat) was then calculated. The fasting body weight of the rat prior to necropsy was used for calculating the relative organ weight.

#### 4.6.7. Histopathology

Organs harvested from all rats were preserved in 10% formalin for histopathology evaluations. Full histopathological examinations were conducted on all collected organs. The histopathological reading, interpretation and reporting were performed by the veterinary pathologist.

### 4.7. Statistical Analysis

The mean value ($\bar{x}$) and standard deviation (σ) were calculated for each variable measured. The statistical analysis was performed using a normality test (Kolmogorov–Smirnov test and Shapiro–Wilk test) and variance homogeneity test (Levene’s test). Normally distributed data were analysed using a parametric test (one-way ANOVA) and data that were significantly different in the parametric test were further analysed using a post hoc test (Tukey HSD (honestly significant difference)). Data that were not normally distributed were analysed using a non-parametric test (Kruskal–Wallis) and data that were significantly different in the non-parametric test were further analysed using another post hoc test (Mann–Whitney U). Analysis of data was carried out using a Microsoft Excel worksheet (Excel, version 2013, Jones, Chicago, IL, USA) and SPSS (SPSS, version 23.0, SPSS Inc., Chicago, IL, USA). Male and female test systems were considered separately for analysis. All the groups were subjected to statistical comparison with the respective control group. Statistically significant differences were recognised at *p* < 0.05.

## 5. Conclusions

The NOAEL for the polyherbal P.SLP formulation is suggested to be more than 500 mg/kg body weight but not exceeding 2000 mg/kg body weight. The early response to exogenous toxicity suggests a study of a longer dosing duration to further investigate the toxicity effects of the formulation.

## Figures and Tables

**Table 1:** Summary of mortality and clinical signs of toxicity in female and male rats.

Female Rats							Male Rats					
Dose (mg/kg) (Group)						Parameters	Dose (mg/kg) (Group)					
0 (Vehicle Control)	125 (Low Dose)	500 (Medium Dose)	2000 (High Dose)	0 (Vehicle Control Satellite)	2000 (High-Dose Satellite)		0 (Vehicle Control)	125 (Low Dose)	500 (Medium Dose)	2000 (High Dose)	0 (Vehicle Control Satellite)	2000 (High-Dose Satellite)
0/5	0/5	0/5	0/5	0/5	1/5	Mortality (died/dosed)	0/5	0/5	0/5	0/5	0/5	0/5
NAD	NAD	NAD	NAD	NAD	NAD	Clinical signs of toxicity	NAD	NAD	NAD	NAD	NAD	NAD
0/5	0/5	0/5	0/5	0/5	0/5	Incidence of clinical signs of toxicity	0/5	0/5	0/5	0/5	0/5	0/5
N/A	N/A	N/A	N/A	N/A	N/A	Onset of clinical signs of toxicity (day)	N/A	N/A	N/A	N/A	N/A	N/A
N/A	N/A	N/A	N/A	N/A	N/A	Duration/severity of clinical signs of toxicity	N/A	N/A	N/A	N/A	N/A	N/A
0/5	0/5	0/5	0/5	0/5	0/5	Incidence of lesions/dosed	0/5	0/5	0/5	0/5	0/5	0/5
N/A	N/A	N/A	N/A	N/A	N/A	Type of lesions	N/A	N/A	N/A	N/A	N/A	N/A
N/A	N/A	N/A	N/A	N/A	N/A	Severity of lesions	N/A	N/A	N/A	N/A	N/A	N/A

Satellite groups were kept for an additional 14 days without treatment to detect delayed occurrence or persistence of, or recovery from, toxic effects. A female rat in the high-dose satellite group died due to technical error in dosing. NAD = no abnormality detected, N/A = not available.

**Table 2:** Summary of functional observations on motor activity and grip strength in female and male rats.

Female Rats							Male Rats					
Dose (mg/kg) (Group)						Parameters	Dose (mg/kg) (Group)					
0 (Vehicle Control)	125 (Low Dose)	500 (Medium Dose)	2000 (High Dose)	0 (Vehicle Control Satellite)	2000 (High-Dose Satellite)		0 (Vehicle Control)	125 (Low Dose)	500 (Medium Dose)	2000 (High Dose)	0 (Vehicle Control Satellite)	2000 (High-Dose Satellite)
1335.40 ± 251.54	1273.00 ± 314.98	1278.00 ± 257.02	1029.80 ± 234.67	1399.80 ± 197.64	1252.00 ± 299.80	General activity	1042.80 ± 306.00	1072.00 ± 183.94	1220.40 ± 61.06	1121.20 ± 159.34	927.60 ± 352.26	790.40 ± 282.33
91.40 ± 19.31	83.80 ± 23.47	83.00 ± 11.68	75.40 ± 11.95	90.00 ± 11.92	95.25 ± 19.60	Stereotyped movement	84.00 ± 19.96	80.80 ± 14.60	96.60 ± 16.86	85.00 ± 12.31	73.60 ± 42.96	55.60 ± 19.83
1244.00 ± 249.15	1189.20 ± 294.44	1195.00 ± 252.36	954.40 ± 226.39	1309.80 ± 190.73	1156.75 ± 283.99	Locomotion	958.80 ± 291.39	991.20 ± 177.56	1123.80 ± 51.80	1036.20 ± 154.56	854.00 ± 310.84	734.80 ± 263.68
555.32 ± 144.31	683.76 ± 164.89	859.74 ± 104.77 *	857.42 ± 155.00 *	828.20 ± 120.30	891.85 ± 119.11	Forelimb grip strength (g)	894.54 ± 143.06	873.68 ± 149.43	1008.78 ± 160.67	943.12 ± 98.40	920.84 ± 140.86	997.30 ± 66.07
1002.08 ± 177.52	955.72 ± 59.88	1013.48 ± 156.98	1027.84 ± 74.99	947.00 ± 158.82	995.08 ± 244.18	Forelimb and hind limb grip strength (g)	1068.46 ± 72.42	1146.04 ± 227.37	1200.40 ± 218.91	1154.40 ± 85.66	1091.04 ± 133.74	1127.62 ± 178.69

Satellite groups were kept for an additional 14 days without treatment to detect delayed occurrence or persistence of, or recovery from, toxic effects. Values are expressed as mean ± standard deviation (*n* = 5). Data for one dead animal were excluded from data analysis, therefore *n* = 4 for female high-dose satellite group. A statistically significant difference was considered at *p* < 0.05 and is denoted with an asterisk (against vehicle control group) in the table. Statistical tests involved were normality, Levene’s, one-way ANOVA, Kruskal–Wallis and/or Tukey HSD (honestly significant difference) tests.

**Table 3:** Percentages of weekly body weight change (%) in female and male rats.

Female Rats							Male Rats					
Dose (mg/kg) (Group)						Weekly Body Weight Change	Dose (mg/kg) (Group)					
0 (Vehicle Control)	125 (Low Dose)	500 (Medium Dose)	2000 (High Dose)	0 (Vehicle Control Satellite)	2000 (High-Dose Satellite)		0 (Vehicle Control)	125 (Low Dose)	500 (Medium Dose)	2000 (High Dose)	0 (Vehicle Control Satellite)	2000 (High-Dose Satellite)
10.41 ± 5.23	8.49 ± 1.65	9.78 ± 2.34	8.89 ± 5.26	6.32 ± 2.02	7.96 ± 2.89	Week 1	12.92 ± 3.67	12.20 ± 1.58	13.10 ± 1.57	13.74 ± 4.27	13.45 ± 2.56	12.33 ± 2.04
2.98 ± 3.22	5.78 ± 1.77	5.31 ± 1.40	5.39 ± 1.90	7.19 ± 3.61	8.97 ± 3.42	Week 2	6.78 ± 2.58	8.34 ± 1.84	9.08 ± 1.97	8.57 ± 0.74	7.61 ± 3.18	7.72 ± 0.69
6.17 ± 5.17	2.03 ± 2.31	4.14 ± 2.63	4.11 ± 3.60	2.95 ± 5.17	1.43 ± 3.23	Week 3	6.36 ± 1.16	6.41 ± 1.15	6.90 ± 1.18	6.23 ± 1.11	7.31 ± 2.14	5.09 ± 2.32
1.81 ± 1.99	3.51 ± 3.47	4.16 ± 3.55	4.39 ± 2.88	3.47 ± 4.22	4.88 ± 2.69	Week 4	2.96 ± 1.11	3.40 ± 1.19	4.39 ± 1.65	3.13 ± 1.07	5.81 ± 0.54	4.64 ± 1.47
N/A	N/A	N/A	N/A	2.73 ± 3.49	1.79 ± 0.67	Week 5	N/A	N/A	N/A	N/A	9.35 ± 9.89	4.81 ± 1.34
N/A	N/A	N/A	N/A	0.78 ± 2.42	3.45 ± 1.02	Week 6	N/A	N/A	N/A	N/A	-0.59 ± 8.95	3.02 ± 1.09

Satellite groups were kept for an additional 14 days without treatment to detect delayed occurrence or persistence of, or recovery from, toxic effects. Values are expressed as mean ± standard deviation (*n* =5). Data for one dead animal were excluded from data analysis, therefore *n* = 4 for female high-dose satellite group. The above values were found to be not statistically significantly different (*p* ≥ 0.05) against the respective control groups. Statistical tests involved were normality, Levene’s, one-way ANOVA and/or Kruskal–Wallis tests. NAD = no abnormality detected, N/A = not available.

**Table 4:** Mean food consumption (g) in female and male rats.

Female Rats							Male Rats					
Dose (mg/kg) (Group)						Weekly Food Consumption	Dose (mg/kg) (Group)					
0 (Vehicle Control)	125 (Low Dose)	500 (Medium Dose)	2000 (High Dose)	0 (Vehicle Control Satellite)	2000 (High-Dose Satellite)		0 (Vehicle Control)	125 (Low Dose)	500 (Medium Dose)	2000 (High Dose)	0 (Vehicle Control Satellite)	2000 (High-Dose Satellite)
118.81 ± 18.27	115.09 ± 10.68	118.75 ± 4.14	118.42 ± 13.48	112.01 ± 6.82	120.69 ± 7.62	Week 1	170.50 ± 15.83	163.38 ± 15.48	171.88 ± 8.13	162.10 ± 14.83	174.30 ± 13.20	170.49 ± 12.48
124.49 ± 12.62	118.91 ± 16.79	128.31 ± 5.78	122.10 ± 12.89	118.91 ± 2.43	130.12 ± 13.76	Week 2	168.53 ± 18.67	166.04 ± 12.46	173.39 ± 9.96	163.51 ± 14.01	176.59 ± 10.77	166.94 ± 14.28
126.75 ± 10.80	116.83 ± 10.71	127.29 ± 2.60	123.48 ± 9.17	118.40 ± 6.19	122.90 ± 9.07	Week 3	170.66 ± 21.76	165.07 ± 16.91	174.58 ± 9.19	158.75 ± 14.62	174.35 ± 12.36	165.23 ± 16.63
110.24 ± 13.49	100.46 ± 8.75	112.10 ± 11.26	108.76 ± 7.43	112.00 ± 4.62	117.33 ± 7.00	Week 4	147.02 ± 17.50	143.65 ± 12.20	151.89 ± 7.31	136.08 ± 16.14	172.85 ± 15.19	156.11 ± 16.95
N/A	N/A	N/A	N/A	116.34 ± 5.88	99.49 ± 5.04	Week 5	N/A	N/A	N/A	N/A	172.37 ± 17.74	165.34 ± 14.94
N/A	N/A	N/A	N/A	126.28 ± 8.37	113.95 ± 6.51	Week 6	N/A	N/A	N/A	N/A	150.87 ± 13.63	153.05 ± 19.47

Satellite groups were kept for an additional 14 days without treatment to detect delayed occurrence or persistence of, or recovery from, toxic effects. Values are expressed as mean ± standard deviation (*n* = 5). Data for one dead animal were excluded from data analysis, therefore *n* = 4 for female high-dose satellite group. The above values were found to be not statistically significantly different (*p* ≥ 0.05) against the respective control groups. Statistical tests involved were normality, Levene’s, one-way ANOVA and/or Kruskal–Wallis tests. N/A = not available.

**Table 5:** Mean water consumption (g) in female and male rats.

Female Rats							Male Rats					
Dose (mg/kg) (Group)						Weekly Water Consumption	Dose (mg/kg) (Group)					
0 (Vehicle Control)	125 (Low Dose)	500 (Medium Dose)	2000 (High Dose)	0 (Vehicle Control Satellite)	2000 (High-Dose Satellite)		0 (Vehicle Control)	125 (Low Dose)	500 (Medium Dose)	2000 (High Dose)	0 (Vehicle Control Satellite)	2000 (High-Dose Satellite)
347.6 ± 99.0	292.2 ± 36.3	340.0 ± 95.5	316.4 ± 85.0	321.8 ± 37.0	275.0 ± 67.4	Week 1	419.0 ± 89.4	371.0 ± 82.1	316.2 ± 34.0	406.4 ± 122.3	358.4 ± 88.9	345.6 ± 36.3
460.6 ± 158.2	325.4 ± 33.3	378.8 ± 88.3	335.2 ± 99.1	347.6 ± 60.4	326.8 ± 62.1	Week 2	409.8 ± 82.8	372.0 ± 80.4	334.8 ± 45.3	423.2 ± 121.3	391.2 ± 96.7	374.4 ± 50.9
497.0 ± 197.8	332.0 ± 42.9	409.4 ± 71.4	342.0 ± 114.7	354.2 ± 66.0	281.3 ± 49.6	Week 3	407.8 ± 111.2	364.0 ± 97.0	298.0 ± 55.5	393.0 ± 131.6	368.0 ± 71.0	366.0 ± 46.2
368.2 ± 129.4	288.2 ± 41.5	400.6 ± 100.9	302.2 ± 69.9	366.4 ± 105.2	292.3 ± 55.5	Week 4	362.8 ± 104.4	330.4 ± 91.0	291.6 ± 34.7	346.0 ± 89.0	354.6 ± 54.6	351.8 ± 66.6
N/A	N/A	N/A	N/A	357.4 ± 117.6	266.8 ± 69.6	Week 5	N/A	N/A	N/A	N/A	349.0 ± 78.2	334.2 ± 47.4
N/A	N/A	N/A	N/A	310.8 ± 85.6	381.5 ± 277.4	Week 6	N/A	N/A	N/A	N/A	317.0 ± 64.8	323.0 ± 78.5

Satellite groups were kept for an additional 14 days without treatment to detect delayed occurrence or persistence of, or recovery from, toxic effects. Values are expressed as mean ± standard deviation (*n* = 5). Data for one dead animal were excluded from data analysis, therefore *n* = 4 for female high-dose satellite group. The above values were found to be not statistically significantly different (*p* ≥ 0.05) against the respective control groups. Statistical tests involved were normality, Levene’s, one-way ANOVA and/or Kruskal–Wallis tests. N/A = not available.

**Table 6:** Haematology findings in female and male rats.

Female Rats							Male Rats					
Dose (mg/kg) (Group)						Parameters	Dose (mg/kg) (Group)					
0 (Vehicle Control)	125 (Low Dose)	500 (Medium Dose)	2000 (High Dose)	0 (Vehicle Control Satellite)	2000 (High-Dose Satellite)		0 (Vehicle Control)	125 (Low Dose)	500 (Medium Dose)	2000 (High Dose)	0 (Vehicle Control Satellite)	2000 (High-Dose Satellite)
33.0 ± 3.1	33.3 ± 1.6	33.3 ± 2.5	32.6 ± 2.5	36.3 ± 2.6	35.8 ± 1.1	**HCT (%)**	36.4 ± 2.2	36.5 ± 3.2	37.6 ± 1.8	35.9 ± 2.3	38.4 ± 2.2	38.4 ± 0.4
12.6 ± 0.5	12.8 ± 0.3	12.9 ± 0.9	12.7 ± 0.6	13.6 ± 0.8	13.6 ± 0.4	**HGB (g/dL)**	13.7 ± 0.6	14.0 ± 0.8	14.2 ± 0.7	13.9 ± 0.7	14.3 ± 0.7	14.5 ± 0.2
6.27 ± 0.48	6.64 ± 0.43	6.68 ± 0.64	6.70 ± 0.48	7.17 ± 0.50	7.22 ± 0.27	**RBC (10^6^ cells/mm)**	7.26 ± 0.51	7.21 ± 0.59	7.61 ± 0.31	7.22 ± 0.40	7.83 ± 0.47	7.90 ± 0.19
3.6 ± 1.3	3.6 ± 1.5	3.0 ± 2.0	4.4 ± 2.8	3.4 ± 1.6	3.3 ± 1.0	**WBC (10^3^ cells/mm)**	7.2 ± 1.0	8.1 ± 1.8	8.5 ± 3.4	7.2 ± 4.0	6.0 ± 1.3	7.4 ± 1.6
1002 ± 123	1132 ± 157	1023 ± 119	1206 ± 119	972 ± 182	1017 ± 133	**PLT (10^3^ cells/mm)**	1124 ± 168	1134 ± 234	1154 ± 188	1316 ± 147	1208 ± 57	1082 ± 134

Satellite groups were kept for an additional 14 days without treatment to detect delayed occurrence or persistence of, or recovery from, toxic effects. Values are expressed as mean ± standard deviation (*n* = 5). Data for one dead animal were excluded from data analysis, therefore *n* = 4 for female high-dose satellite group. The above values were found to be not statistically significantly different (*p* ≥ 0.05) against the respective control groups. Statistical tests involved were normality, Levene’s, one-way ANOVA, Kruskal–Wallis and/or Tukey HSD tests. HCT = haematocrit, HGB = haemoglobin, RBC = red blood cells, WBC = white blood cells, PLT = platelet.

**Table 7:** Clinical biochemistry liver profile parameter in female and male rats.

Female Rats							Male Rats					
Dose (mg/kg) (Group)						Parameters	Dose (mg/kg) (Group)					
0 (Vehicle Control)	125 (Low Dose)	500 (Medium Dose)	2000 (High Dose)	0 (Vehicle Control Satellite)	2000 (High-Dose Satellite)		0 (Vehicle Control)	125 (Low Dose)	500 (Medium Dose)	2000 (High Dose)	0 (Vehicle Control Satellite)	2000 (High-Dose Satellite)
						**Liver Profile**						
62.3 *±* 2.1	62.8 *±* 1.8	60.4 *±* 2.3	60.0 *±* 2.9	62.0 *±* 1.0	62.5 *±* 3.3	**Total protein (g/L)**	60.2 *±* 3.1	65.6 *±* 3.9	61.8 *±* 4.2	60.4 *±* 2.1	61.0 *±* 3.4	60.4 *±* 2.6
14.7 *±* 0.5	15.0 *±* 0.0	13.8 *±* 1.3	14.0 *±* 1.2	14.4 *±* 0.9	15.0 *±* 0.8	**Albumin (g/L)**	13.2 *±* 1.3	13.8 *±* 0.8	13.2 *±* 0.8	12.6 *±* 0.5	12.8 *±* 0.4	12.6 *±* 0.5
32.1 *±* 5.9	35.0 *±* 8.5	34.2 *±* 4.3	28.8 *±* 5.9	34.4 *±* 5.7	30.3 *±* 3.7	**ALT (U/L)**	40.2 *±* 10.1	34.4 *±* 5.8	32.8 *±* 6.1	31.8 *±* 5.1	37.8 *±* 6.2	34.4 *±* 7.4
83.2 *±* 14.2	79.0 *±* 23.5	67.2 *±* 22.3	51.4 *±* 9.8	67.4 *±* 22.5	54.8 *±* 12.7	**ALP (U/L)**	127.4 *±* 24.8	108.20 ± 14.8	119.0 *±* 25.1	93.6 *±* 36.8	91.0 *±* 9.2	90.6 *±* 7.3
109.8 *±* 6.0	96.8 *±* 14.8	100.2 *±* 9.7	104.6 *±* 23.2	81.2 *±* 11.7	78.8 *±* 11.7	**AST (U/L)**	80.3 *±* 13.0	74.6 *±* 17.1	74.8 *±* 7.0	76.6 *±* 9.0	70.8 *±* 10.6	62.0 *±* 3.5

Satellite groups were kept for an additional 14 days without treatment to detect delayed occurrence or persistence of, or recovery from, toxic effects. Values are expressed as mean ± standard deviation (*n* = 5). Data for one dead animal were excluded from data analysis, therefore *n* = 4 for female high-dose satellite group. The above values were found to be not statistically significantly different (*p* ≥ 0.05) against the respective control groups. Statistical tests involved were normality, Levene’s, one-way ANOVA, Kruskal–Wallis, Tukey HSD and/or Mann–Whitney U tests. ALT = alanine amino-transferase, ALP = alkaline phosphatase, AST = aspartate amino-transferase.

**Table 8:** Clinical biochemistry renal and lipid profile parameter on female and male rats.

Female Rats							Male Rats					
Dose (mg/kg) (Group)						Parameters	Dose (mg/kg) (Group)					
0 (Vehicle Control)	125 (Low Dose)	500 (Medium Dose)	2000 (High Dose)	0 (Vehicle Control Satellite)	2000 (High-Dose Satellite)		0 (Vehicle Control)	125 (Low Dose)	500 (Medium Dose)	2000 (High Dose)	0 (Vehicle Control Satellite)	2000 (High-Dose Satellite)
						**Renal Profile**						
4.1 *±* 0.3	3.8 *±* 0.2	4.2 *±* 0.5	4.0 *±* 0.4	3.9 *±* 0.6	3.7 *±* 0.2	**Potassium (mmol/L)**	4.0 *±* 0.3	4.4 *±* 0.4	4.1 *±* 0.3	4.5 *±* 0.2	4.0 *±* 0.4	4.0 *±* 0.3
38.4 *±* 7.6	33.8 *±* 2.7	39.0 *±* 2.5	35.2 *±* 5.4	39.4 *±* 7.4	41.5 *±* 9.3	**Creatinine (µmol/L)**	30.4 *±* 3.2	32.2 *±* 3.6	34.0 *±* 7.0	27.2 *±* 6.3	37.0 *±* 6.1	32.4 *±* 5.7
7.1 *±* 0.7	6.7 *±* 0.7	7.5 *±* 1.0	6.1 *±* 1.4	6.6 *±* 1.1	7.2 *±* 0.7	**Urea (mmol/L)**	6.7 *±* 0.4	6.9 *±* 0.9	6.9 *±* 0.7	6.6 *±* 1.1	6.5 *±* 1.1	7.1 *±* 1.3
94.3 *±* 9.1	78.6 *±* 30.5	111.8 *±* 22.0	95.2 *±* 25.8	77.8 *±* 27.5	98.8 *±* 35.7	**Uric acid (µmol/L)**	77.2 *±* 5.8	78.80 *±* 18.1	71.0 *±* 28.2	68.6 *±* 14.3	69.6 *±* 15.7	82.4 *±* 48.6
						**Lipid Profile**						
1.9 *±* 0.2	2.1 *±* 0.4	2.1 *±* 0.2	1.7 *±* 0.1	2.1 *±* 0.3	2.0 *±* 0.2	**Total cholesterol (mmol/L)**	1.7 *±* 0.2	2.3 *±* 0.4	1.8 *±* 0.2	1.4 *±* 0.3	2.3 *±* 0.1	2.1 *±* 0.3
0.4 *±* 0.1	0.5 *±* 0.1	0.4 *±* 0.1	0.4 *±* 0.2	0.7 *±* 0.2	0.5 *±* 0.2	**Triglyceride (mmol/L)**	0.7 *±* 0.2	1.0 *±* 0.4	1.0 *±* 0.3	1.0 *±* 0.3	0.9 *±* 0.2	0.9 *±* 0.3
6.71 *±* 0.40	6.70 *±* 0.81	6.87 *±* 0.71	7.26 *±* 0.66	8.42 *±* 1.13	7.45 *±* 0.78	**Glucose (mmol/L)**	8.70 *±* 0.56	8.58 *±* 0.79	8.96 *±* 0.90	9.64 *±* 1.72	8.57 *±* 0.74	9.31 *±* 2.05

Satellite groups were kept for an additional 14 days without treatment to detect delayed occurrence or persistence of, or recovery from, toxic effects. Values are expressed as mean ± standard deviation (*n* = 5). Data for one dead animal were excluded from data analysis, therefore *n* = 4 for female high-dose satellite group. The above values were found to be not statistically significantly different (*p* ≥ 0.05) against the respective control groups. Statistical tests involved were normality, Levene’s, one-way ANOVA, Kruskal–Wallis, Tukey HSD and/or Mann–Whitney U tests.

**Table 9:** Summary of gross pathology findings in female and male rats.

Female Rats							Male Rats					
Dose (mg/kg) (Group)						Organs	Dose (mg/kg) (Group)					
0 (Vehicle Control)	125 (Low Dose)	500 (Medium Dose)	2000 (High Dose)	0 (Vehicle Control Satellite)	2000 (High-Dose Satellite)		0 (Vehicle Control)	125 (Low Dose)	500 (Medium Dose)	2000 (High Dose)	0 (Vehicle Control Satellite)	2000 (High-Dose Satellite)
5/5	4/5	5/5	5/5	5/5	4/5	**Lung**	4/5	5/5	4/5	5/5	5/5	4/5
0/5	0/5	0/5	0/5	0/5	0/5	**Heart**	0/5	0/5	0/5	0/5	0/5	0/5
0/5	0/5	0/5	0/5	0/5	0/5	**Spleen**	0/5	0/5	0/5	0/5	0/5	0/5
0/5	0/5	0/5	0/5	0/5	0/5	**Stomach**	0/5	0/5	0/5	0/5	0/5	0/5
1/5	3/5	3/5	3/5	0/5	0/5	**IT**	0/5	2/5	1/5	1/5	0/5	5/5
3/5	2/5	3/5	3/5	1/5	2/5	**Liver**	1/5	2/5	2/5	1/5	1/5	1/5
0/5	0/5	0/5	0/5	0/5	0/5	**Kidneys**	0/5	0/5	0/5	0/5	0/5	5/5
0/5	0/5	0/5	0/5	0/5	0/5	**Adrenals**	0/5	0/5	0/5	0/5	0/5	0/5
0/5	0/5	0/5	1/5	0/5	0/5	**Ovaries**	N/A	N/A	N/A	N/A	N/A	N/A
0/5	0/5	1/5	0/5	0/5	1/5	**Uterine horn**	N/A	N/A	N/A	N/A	N/A	N/A
N/A	N/A	N/A	N/A	N/A	N/A	**Testes**	0/5	0/5	0/5	0/5	0/5	0/5

Satellite groups were kept for an additional 14 days without treatment to detect delayed occurrence or persistence of, or recovery from, toxic effects. Values indicate the number of rats with organ(s) that showed macroscopic lesions during gross examination but were not necessarily P.SLP formulation related. IT = intestinal tract. N/A = not available.

**Table 10:** Relative organ weight in female and male rats.

Female Rats							Male Rats					
Dose (mg/kg) (Group)						Organs	Dose (mg/kg) (Group)					
0 (Vehicle Control)	125 (Low Dose)	500 (Medium Dose)	2000 (High Dose)	0 (Vehicle Control Satellite)	2000 (High-Dose Satellite)		0 (Vehicle Control)	125 (Low Dose)	500 (Medium Dose)	2000 (High Dose)	0 (Vehicle Control Satellite)	2000 (High-Dose Satellite)
0.4444 *±* 0.0454	0.4358 *±* 0.0209	0.4191 *±* 0.0330	0.3871 *±* 0.0310	0.3905 *±* 0.0135	0.3893 *±* 0.0216	**Lung**	0.3399 *±* 0.0456	0.3528 *±* 0.0203	0.3135 *±* 0.04	0.3486 *±* 0.0432	0.3057 *±* 0.0318	0.3459 *±* 0.0890
0.2890 *±* 0.0307	0.2850 *±* 0.0252	0.2876 *±* 0.0132	0.2930 *±* 0.0219	0.2729 *±* 0.0158	0.2769 *±* 0.0104	**Heart**	0.2494 *±* 0.0331	0.2639 *±* 0.0231	0.2559 *±* 0.0132	0.2539 *±* 0.0357	0.2297 *±* 0.0185	0.2390 *±* 0.0229
0.2332 *±* 0.0281	0.2292 *±* 0.0246	0.2282 *±* 0.0304	0.2180 *±* 0.0196	0.2028 *±* 0.0126	0.2000 *±* 0.0139	**Spleen**	0.2063 *±* 0.0331	0.2052 *±* 0.0244	0.1816 *±* 0.0227	0.1829 *±* 0.0124	0.1731 *±* 0.0183	0.1962 *±* 0.0205
0.5471 *±* 0.0688	0.5849 *±* 0.0919	0.5867 *±* 0.0651	0.5605 *±* 0.0247	0.5328 *±* 0.0269	0.5273 *±* 0.0155	**Stomach**	0.4714 *±* 0.0494	0.4856 *±* 0.0490	0.4743 *±* 0.0329	0.4823 *±* 0.0618	0.4533 *±* 0.0366	0.4418 *±* 0.0263
2.6132 *±* 0.6347	2.6427 *±* 0.5049	2.6492 *±* 0.7822	2.5011 *±* 0.3024	2.3388 *±* 0.4279	2.1914 *±* 0.1792	**IT**	1.8803 *±* 0.3418	2.1921 *±* 0.2580	2.0550 *±* 0.2891	2.0597 *±* 0.2814	2.0162 *±* 0.2676	1.7278 *±* 0.0613
2.7278 *±* 0.1933	2.5790 *±* 0.1221	2.5379 *±* 0.0668	2.6613 *±* 0.0964	2.4010 *±* 0.1312	2.5351 *±* 0.0276	**Liver**	2.9989 *±* 0.4124	3.1014 *±* 0.3272	2.8786 *±* 0.1110	3.0926 *±* 0.1527	2.7864 *±* 0.2314	2.9335 *±* 0.1799
0.3155 *±* 0.0174	0.3255 *±* 0.0291	0.3051 *±* 0.0061	0.3325 *±* 0.0233	0.2904 *±* 0.0300	0.3216 *±* 0.0228	**Kidney Right**	0.3248 *±* 0.0252	0.3339 *±* 0.0269	0.3082 *±* 0.0085	0.3238 *±* 0.0135	0.3083 *±* 0.0192	0.3318 *±* 0.0355
0.3172 *±* 0.0171	0.3254 *±* 0.0198	0.2910 *±* 0.0119	0.3199 *±* 0.0316	0.2836 *±* 0.0300	0.3147 *±* 0.0230	**Kidney Left**	0.3252 *±* 0.0204	0.3283 *±* 0.0264	0.3111 *±* 0.0129	0.3171 *±* 0.0251	0.3034 *±* 0.0265	0.3270 *±* 0.0371

Satellite groups were kept for an additional 14 days without treatment to detect delayed occurrence or persistence of, or recovery from, toxic effects. Values are expressed as mean ± standard deviation (*n* = 5). Data for one dead animal were excluded from data analysis, therefore *n* = 4 for female high-dose satellite group. The above values were found to be not statistically significantly different (*p* ≥ 0.05) against the respective control groups. Statistical tests involved were normality, Levene’s, one-way ANOVA, Kruskal–Wallis and/or Tukey HSD tests. IT = intestinal tract.

**Table 11:** Relative organ weight (adrenals and reproductive organs) in female and male rats.

Female Rats							Male Rats					
Dose (mg/kg) (Group)						Organs	Dose (mg/kg) (Group)					
0 (Vehicle Control)	125 (Low Dose)	500 (Medium Dose)	2000 (High Dose)	0 (Vehicle Control Satellite)	2000 (High-Dose Satellite)		0 (Vehicle Control)	125 (Low Dose)	500 (Medium Dose)	2000 (High Dose)	0 (Vehicle Control Satellite)	2000 (High-Dose Satellite)
0.0152 *±* 0.0036	0.0137 *±* 0.0012	0.0157 *±* 0.0024	0.0134 *±* 0.0044	0.0130 *±* 0.0013	0.0130 *±* 0.0010	**Adrenal Right**	0.0083 *±* 0.0016	0.0086 *±* 0.0012	0.0077 *±* 0.0009	0.0077 *±* 0.0015	0.0065 *±* 0.0013	0.0071 *±* 0.0012
0.0168 *±* 0.0043	0.0148 *±* 0.0012	0.0149 *±* 0.0023	0.0161 *±* 0.0023	0.0143 *±* 0.0018	0.0130 *±* 0.0027	**Adrenal Left**	0.0082 *±* 0.0010	0.0091 *±* 0.0014	0.0084 *±* 0.0007	0.0086 *±* 0.0012	0.0077 *±* 0.0013	0.0081 *±* 0.0007
0.0275 *±* 0.0035	0.0252 *±* 0.0083	0.0207 *±* 0.0042	0.0223 *±* 0.0065	0.0196 *±* 0.0024	0.0169 *±* 0.0028	**Ovary Right**	N/A	N/A	N/A	N/A	N/A	N/A
0.0256 *±* 0.0029	0.0188 *±* 0.0036 *	0.0166 *±* 0.0033 *	0.0202 *±* 0.0040	0.0205 *±* 0.0019	0.0183 *±* 0.0043 ^#^	**Ovary Left**	N/A	N/A	N/A	N/A	N/A	N/A
0.0624 *±* 0.0864	0.0718 *±* 0.1002	0.1533 *±* 0.0964	0.1619 *±* 0.0969	0.1732 *±* 0.0535	0.2122 *±* 0.0421	**Uterine Horn**	N/A	N/A	N/A	N/A	N/A	N/A
N/A	N/A	N/A	N/A	N/A	N/A	**Testes Right**	0.4504 *±* 0.0596	0.4121 *±* 0.0309	0.4068 *±* 0.0361	0.4320 *±* 0.0251	0.3924 *±* 0.0289	0.4110 *±* 0.0316
N/A	N/A	N/A	N/A	N/A	N/A	**Testes Left**	0.4595 *±* 0.0874	0.4096 *±* 0.0272	0.4099 *±* 0.0366	0.4290 *±* 0.0238	0.3936 *±* 0.0303	0.4156 *±* 0.0480

Satellite groups were kept for an additional 14 days without treatment to detect delayed occurrence or persistence of, or recovery from, toxic effects. Values are expressed as mean ± standard deviation (*n* = 5). Data for one dead animal were excluded from data analysis, therefore *n* = 4 for female high-dose satellite group. Statistically significant difference was considered at *p* < 0.05 and is denoted with an asterisk (significantly different against female vehicle control group) and a hash mark (significantly different against female vehicle control satellite group) in the table. Statistical tests involved were normality, Levene’s, one-way ANOVA, Kruskal–Wallis and/or Tukey HSD tests.

**Table 12:** Summary of microscopic findings in female and male rats.

Female Rats							Male Rats					
Dose (mg/kg) (Group)						Organs	Dose (mg/kg) (Group)					
0 (Vehicle Control)	125 (Low Dose)	500 (Medium Dose)	2000 (High Dose)	0 (Vehicle Control Satellite)	2000 (High-Dose Satellite)		0 (Vehicle Control)	125 (Low Dose)	500 (Medium Dose)	2000 (High Dose)	0 (Vehicle Control Satellite)	2000 (High-Dose Satellite)
0/5	5/5	4/5	4/5	5/5	5/5	**Lung**	2/5	5/5	5/5	5/5	5/5	5/5
0/5	0/5	0/5	5/5	0/5	5/5	**Heart**	0/5	0/5	0/5	5/5	0/5	5/5
0/5	2/5	0/5	1/5	0/5	0/5	**Spleen**	0/5	0/5	0/5	1/5	0/5	0/5
0/5	0/5	0/5	4/5	0/5	5/5	**Stomach**	0/5	0/5	0/5	5/5	0/5	5/5
0/5	3/5	0/5	4/5	0/5	5/5	**IT**	0/5	2/5	1/5	5/5	0/5	5/5
0/5	5/5	3/5	4/5	0/5	5/5	**Liver**	0/5	2/5	1/5	5/5	0/5	5/5
0/5	5/5	3/5	4/5	0/5	5/5	**Kidneys**	0/5	0/5	0/5	5/5	0/5	5/5
0/5	0/5	0/5	1/5	0/5	0/5	**Adrenals**	0/5	0/5	0/5	0/5	0/5	0/5
0/5	0/5	0/5	1/5	0/5	0/5	**Ovaries**	N/A	N/A	N/A	N/A	N/A	N/A
0/5	0/5	0/5	0/5	0/5	0/5	**Uterine Horn**	N/A	N/A	N/A	N/A	N/A	N/A
N/A	N/A	N/A	N/A	N/A	N/A	**Testes**	0/5	0/5	0/5	0/5	0/5	0/5

Satellite groups were kept for an additional 14 days without treatment to detect delayed occurrence or persistence of, or recovery from, toxic effects. Values indicate the number of rats with organ(s) that showed microscopic lesions but were not necessarily P.SLP formulation related. IT = intestinal tract.

## Data Availability

Please refer to suggested Data Availability Statements in section “MDPI Research Data Policies” at https://www.mdpi.com/ethics.
